# Role of Endoglin (CD105) in the Progression of Hepatocellular Carcinoma and Anti-Angiogenic Therapy

**DOI:** 10.3390/ijms19123887

**Published:** 2018-12-05

**Authors:** Aldona Kasprzak, Agnieszka Adamek

**Affiliations:** 1 Department of Histology and Embryology, University of Medical Sciences, Poznań 60-781, Poland; 2 Department of Infectious Diseases, Hepatology and Acquired Immunodeficiencies, University of Medical Sciences, Poznań 61-285, Poland; agnieszkaadamek@ump.edu.pl

**Keywords:** hepatocellular carcinoma, tumour microvasculature, TGF-β auxiliary receptors, Endoglin (CD105), MVD-CD105 score

## Abstract

The liver is perfused by both arterial and venous blood, with a resulting abnormal microenvironment selecting for more-aggressive malignancies. Hepatocellular carcinoma (HCC) is the most frequent primary liver cancer, the sixth most common cancer globally, and the third leading cause of cancer-related mortality worldwide. HCC is characterized by its hypervascularization. Improving the efficiency of anti-angiogenic treatment and mitigation of anti-angiogenic drug resistance are the top priorities in the development of non-surgical HCC therapies. Endoglin (CD105), a transmembrane glycoprotein, is one of the transforming growth factor β (TGF-β) co-receptors. Involvement of that protein in angiogenesis of solid tumours is well documented. Endoglin is a marker of activated endothelial cells (ECs), and is preferentially expressed in the angiogenic endothelium of solid tumours, including HCC. HCC is associated with changes in CD105-positive ECs within and around the tumour. The large spectrum of endoglin effects in the liver is cell-type- and HCC- stage-specific. High expression of endoglin in non-tumour tissue suggests that this microenvironment might play an especially important role in the progression of HCC. Evaluation of tissue expression, as well as serum concentrations of this glycoprotein in HCC, tends to confirm its role as an important biomarker in HCC diagnosis and prognosis. The role of endoglin in liver fibrosis and HCC progression also makes it an attractive therapeutic target. Despite these facts, the exact molecular mechanisms of endoglin functioning in hepatocarcinogenesis are still poorly understood. This review summarizes the current data concerning the role and signalling pathways of endoglin in hepatocellular carcinoma development and progression, and provides an overview of the strategies available for a specific targeting of CD105 in anti-angiogenic therapy in HCC.

## 1. Introduction

Hepatocellular carcinoma (HCC) is the most frequent primary liver cancer, the sixth most common cancer globally, and the third leading cause of cancer-related mortality in both sexes worldwide, with increasing incidence and mortality [1,2]. Molecular mechanisms of hepatocellular carcinogenesis may vary depending on different factors, which is why many mechanisms have been associated with this tumour [2,3]. 

HCC is one of the highly vascularized solid tumours, with angiogenesis playing an important role in its development, growth rate, and prognosis [4,5]. Many cytotoxic chemotherapeutic agents have been tested in patients with advanced disease, with disappointing outcomes and poor tolerance. Therefore, no standard systemic therapy emerged until the approval of sorafenib in 2007 [6,7]. Sorafenib is a small multi-tyrosine kinase inhibitor that blocks the activity of Raf kinase, the Vascular Endothelial Growth Factor Receptor (VEGF-R), and the Platelet-Derived Growth Factor Receptor (PDGF-R) [2,6,8]. Some trials have used other anti-angiogenic drugs to target multiple tyrosine kinase targets, mainly combined with sorafenib. In advanced HCC, the standard life-extending drugs, apart from sorafenib, are lenvatinib (which was non-inferior to sorafenib in phase III trials), and regorafenib (which was the only drug that demonstrated survival benefit as a second-line treatment) [2,7]. However, the side effects of anti-angiogenic treatments are commonly described. They include endothelial cells (ECs) drug resistance and drug-induced hypoxia in the tumour region, which may even increase the invasiveness of cancer cells and hasten the metastasis [9]. Hence, it seems important to conduct a complex analysis of the molecular mechanisms of HCC angiogenesis, as well as the role of less studied factors involved in this process.

Due to the observation that endoglin (CD105) is selectively expressed (or overexpressed) in activated vascular ECs in tumours (including HCC), it was hypothesized that it can also be a useful target for vascular-targeted anti-angiogenic therapy [10]. The commonly suggested role of CD105 in carcinogenesis is based on clinical studies, as well as in vitro and animal model experiments. The results of said research indicate the potential role of CD105 in liver fibrosis [11,12,13] and hepatocellular carcinoma progression [12,14,15,16,17,18,19,20,21,22]. However, the observations concerning quantitative endoglin expression and its prognostic role in HCC are not coherent. Some report that tissue expression in ECs of tumour tissue, as well as soluble endoglin (Sol-ENG) serum levels, positively correlate with more advanced clinical stage and/or poor prognosis [14,15,16,17,18,19]. Other studies report higher tissue expression of CD105 in ECs of non-tumour tissue, in comparison with tumours and/or control liver, with correlations of clinical staging and/or HCC prognosis visible only for that location [12,20,21,22]. 

The role of TGF-β systems (including endoglin) in carcinogenesis of solid tumours was well described in many works. However, none of them focused on HCC in particular [23]. The molecular mechanisms of HCC involving endoglin described in the literature connect this marker with both tumour angiogenesis [4,5] and liver fibrosis [11,12,13], while the autocrine/paracrine mechanisms of action of endoglin, produced by tumour cells, are poorly recognized [23,24]. 

Currently, most works describing the role of factors stimulating the angiogenic process in human cancers (including HCC) were focused on VEGF [5,8,25]. This is understandable, as this protein appears to be the most critical angiogenic factor, and the blockade of VEGF-mediated pathways (by e.g., sorafenib) suppresses carcinogenesis and angiogenesis in HCC [5,7,8]. However, adverse effects of anti-VEGF therapy (e.g., the consequences of damage to not only the tumour vessels, but also healthy ones, mechanisms of resistance to VEGF blockade, etc.) are also often described [5,7]. One way to overcome these limitations is the search for new forms of anti-angiogenic therapy of advanced HCC, using different approaches than those targeting VEGF [7,10].

To our knowledge, there is not yet a review that summarizes the results of basic and clinical studies that investigate the role of endoglin in HCC progression. Hence, an attempt to fill this gap was made in this manuscript, choosing endoglin as its focus for several reasons: (1) expression of that glycoprotein represents the proliferation status of liver sinusoidal ECs (LSECs), which are key in angiogenesis of the HCC microenvironment; (2) endoglin seems to be a better quantitative marker of microvessels density (MVD) compared to other microvessel markers e.g., CD34 [15], which allows for more credible evaluation of HCC angiogenesis in humans; (3) the results of studies on the role of endoglin in pathogenesis and clinical treatment of HCC are not coherent, with the causes of that divergence currently unexplained [14,15,16,17,18,19,20,21,22]; (4) there is currently no meta-analysis of the diagnostic-prognostic role of that marker in HCC; (5) while endoglin is a promising target of anti-angiogenic therapy of solid tumours, there are not many clinical trials of its application in advanced HCC [10]; and (6) comparison of the available results of studies on the role of endoglin in HCC, as well as the function of that glycoprotein in liver physiology, seems to be of great practical (diagnostics, prognostics, therapy) and basic scientific importance.

Thus, in this review, we have summarized recent studies of endoglin functions and related signalling pathways as a diagnostic-prognostic biomarker of HCC and a potential molecular target of anti-angiogenic therapy. The available data on the role of endoglin in angiogenesis were updated, with an attempt to explain the mechanisms of CD105 action in the CD105 microenvironment of HCC in humans. In the review of works describing anti-angiogenic therapies, we have particularly focused on manuscripts that analyse endoglin as a target, and which could have applications in HCC therapy. 

## 2. From Tumor-Angiogenesis Factor (TAF) through Vascular Endothelial Growth Factor (VEGF) to Endoglin

Angiogenesis, a multi-stage process of blood vessel formation, is closely associated with neoplasia. The vascular network plays a major role in the creation of an immunosuppressive environment that allows tumours to “escape” from the immune system effects. New blood vessels are a proxy that facilitates cancer cell expansion, as well as tumour microenvironment maintenance [25,26]. On the tissue level, a decisive role in vessel migration and vasculogenesis is played by vascular endothelial cells, with inhibition of their proliferation making it possible to impede primary tumour growth and metastasis [25,26,27]. 

The role of endoglin in tumour-associated angiogenesis, as well as recent tests of anti-endoglin therapies, need to be briefly related to two other pro-angiogenic factors which are closely associated with ECs activation. Historically, the first recognised factor of this type was tumour-angiogenesis factor (TAF), discovered in the 1970s [27]. The isolation of TAF can be called a milestone which, along with fast development of biotechnology, resulted in a cascade of new angiogenic factor discoveries, as well as a large improvement in cancer knowledge. 

The second factor was VEGF, the discovery of which also proved to be a scientific and therapeutic breakthrough, as it marked the beginning of a new era in anti-angiogenic therapy of solid tumours in humans (including HCC) [2,5,6,7]. Currently, the number of works describing the role of VEGF is more than 70,000, with more than a 1000 literature positions related to VEGF and HCC, according to the PubMed database.

The bases of current knowledge of solid tumour angiogenesis (including HCC) were undoubtedly laid by Judah Folkman, who first emphasized the importance of tumour vascularity for tumour growth at the beginning of the 1970s [25,27]. Called the “Father of Angiogenesis”, in 1971, he isolated and described the soluble mitogenic factor influencing vascular ECs. The fraction that exhibited the strongest angiogenic activity, with a molecular weight of about 10 kDa, was subsequently called “tumour-angiogenesis factor” (TAF) [27]. Inhibition of this angiogenic factor impaired angiogenesis of solid tumours (including hepatoblastoma), causing their shrinking to just a few millimetres in size [27]. This researcher was also a creator of the „angiogenic switch” concept, describing a process involving the increase in pro-angiogenic factors concentration, as well as inhibition of angiogenesis inhibitors synthesis, that precedes the sprouting of new blood vessels. Folkman also described two phases of tumour growth, i.e., the avascular and vascular phase, as well as discovering the first anti-angiogenic factor (the neonatal cartilage) which impaired tumour growth through the inhibition of ECs proliferation [28]. The research by Folkman, followed by a growing team of researchers, has resulted in the discovery of further potent (and more specific) angiogenic proteins and/or angiogenesis inhibitors. 

Thus, another more specific pro-angiogenic factor was first discovered by the group of Senger et al. and initially called Vascular Permeability Factor (VPF) (currently VEGF). This factor was isolated from both the culture medium and ascites fluid of one tumour, the guinea pig line 10 hepatocarcinoma as a protein of 34–42 kDa in size, which differed from the permeability factors that were known at the time [29]. Currently, VEGF is the best studied pro-angiogenic factor in both physiological and pathological angiogenesis (including solid tumours) [5,25]. When the tumours grow to 0.2–2.0 mm in diameter, they become hypoxic and demonstrated limited growth in the absence of angiogenesis. During the angiogenic switch, pro-angiogenic factors predominate and result in angiogenesis and tumour progression [26,27,30,31,32,33]. Increased production of VEGF follows the up-regulation of the hypoxia-inducible transcription factor (HIF) [33]. Several members of the VEGF family have been described, namely the VEGF-A, B, C, D, E, and placental growth factor (PlGF) [34]. These factors bind specific receptors present on EC surface (VEGFR-1, VEGFR-2, VEGFR-3, neuropilin-1 and -2), which dimerise and activate the intracellular tyrosine kinases, conducting the angiogenesis promoting signals [35]. Autocrine pathway of tumour cell activation is also possible, as VEGF receptor presence was also described in cultured HCC cells [8,36]. Tumour cells are not the only source of VEGF, with this factor also being expressed by tumour-associated stromal cells [37] and alternatively-activated macrophages (M2) [25]. VEGF is the most well-studied angiogenic factor in the HCC, initiating several signalling pathways, resulting in proliferation, migration, and invasion of ECs [5]. 

Another protein linked to the vascular endothelium was discovered in the 1990s. Initially called the 44G4 antigen, it was composed of two subunits of 95 kDa molecular weight, linked by disulphide bond(s) [38]. Today, it is known that this antigen, officially named endoglin, is a major glycoprotein of the human vascular endothelium [23,24]. Its role in vascular EC adhesion was first detected using 44G4 monoclonal antibody (mAb), produced against a human pre-B leukemic cell line. Immunofluorescence staining of ECs confirmed that mAb 44G4 recognized a surface membrane component of vascular endothelium. Endoglin is a glycoprotein expressed constitutively, in high quantities, on human ECs of the capillaries, arterioles, and venules in a variety of tissues [38]. The research on cardiovascular system development in mice noted the elevated expression of endoglin in capillaries, as compared to veins or arteries [39]. Endoglin is a membrane marker of low expression in resting ECs, which rises significantly in proliferating angiogenic ECs [40,41,42,43]. Identification of LSECs confirmed the CD105 as a common EC marker. These cells play a key role in the initiation and progression of chronic liver disease, through sinusoidal capillarization, angiogenesis, angiocrine signals, and vasoconstriction. LSECs are also important in HCC development and progression [44]. Endoglin was assigned the cluster of differentiation number of 105 at the Fifth International Workshop on Human Leukocyte Differentiation Antigens and thus is also known as CD105 (CD105 antigen) [41].

Similar to VEGF, endoglin expression is also detected on cellular membranes and in the cytoplasm of non-endothelial cells of normal and malignant tissues [23,41,45,46,47,48]. Interestingly, in HCC samples, 35% of normal hepatocytes in non-cancerous areas and 55% of cancer cells expressed cytoplasmic CD105 [45]. Various in vitro cell lines, including those of normal hepatocytes [49], HCC cancer cells [16,50], as well as undifferentiated and differentiated adult-derived human liver stem/progenitor cells [49,51], human adipose-derived stem cells [52], and hepatic perivascular mesenchymal stem cells [48,53] are also characterised by endoglin expression of varying levels.

In summary, all three of the described factors are glycoproteins that share, above all, their endothelial cell-specific mitogenic activities (pro-angiogenic factors). This activity mostly occurs in conditions of long-term tissue hypoxia, which accompanies neoplasia. 

Research articles suggest that these proteins tightly cooperate in the angiogenic process, taking complex responsibility for the quantitative and qualitative changes of the tumour vasculature. The change of blood vessel phenotype involves the appearance of new endothelial tumour markers, e.g., VEGFRs, and/or endoglin. While the signalling pathways of VEGF and endoglin differ, the final effect of the action of both of these factors is pro-angiogenic signalling. Detailed mechanisms of endoglin as a membranous EC marker were described in the further parts of this manuscript. 

All three of the mentioned proteins can be produced by different cell types, including: (1) tumour cells (TAF, VEGF, endoglin in a lower extent), (2) endothelial cells (VEGFRs, endoglin), (3) numerous non-endothelial cells (all three proteins). The subcellular location of these proteins also differs; VEGF is largely present in the cytoplasm, while endoglin is mostly a membranous protein. It is worth noting that increased endoglin expression can be observed in proliferating tumour ECs, with only small expression in resting ECs, which might be an advantage in the choice of anti-endoglin therapy. 

## 3. Angiogenesis in HCC

HCC is one of the most vascular solid tumours, in which angiogenesis plays an important role [4,5,54,55]. The best-known molecular pathway driving tumour vascularization (including HCC) is the hypoxia-adaptation mechanism. Basic studies and clinical research over recent years have suggested that the process of neoangiogenesis involves the bone marrow-derived endothelial progenitor cells (BM-EPCs), as well as ECs, co-opted from surrounding vessels [30,31]. The angiogenic process is initiated on various stages of carcinogenesis, including dysplastic nodules (DN), regenerative nodules (RN), and mature HCC [12,56,57,58,59]. HCC is mainly supplied by hepatic arteries. Meanwhile, normal liver parenchyma, RN, and DN are mainly supplied by the portal vein. It was shown that abnormal arterial supply is associated with the differentiation of the HCC [55]. Stimulation of angiogenesis causes changes in liver architecture. The vascular abnormalities correlate with the progression of cancerous changes and can be a prognostic factor [57]. Evaluation of intratumoral MVD with the use of CD34 (a marker of active LSECs, not present on normal sinusoidal ECs) immunostaining presented a positive correlation with tumour size, poorer differentiation, and portal invasion, while correlating negatively with overall survival (OS) [54]. The high MVD-CD34 score was predictive of early postresection recurrence, and was the only significant predictive factor of disease-free survival (DFS) in patients with HCC ≤ 5 cm [4]. 

HCC is characterized by its hypervasculature resulting from higher expression of angiogenesis promoting factors, especially VEGF and VEGF-R, but also other pro-angiogenic factors, such as angiopoietin 2, PDGF, EGF, FGF, TGF-α, and endoglin [55,57,59,60,61]. A critical role in vascular change induction in HCC, similarly to other solid tumours, is played by VEGF, which interacts with various hepatic cell types, mainly ECs, hepatic stellate cells (HSCs), EPCs, and hemangiocytes [5].

The liver is perfused by both arterial and venous blood, with the resulting abnormal microenvironment selecting for more-aggressive malignancies [62]. Liver sinusoids have a dual blood supply, receiving blood from the portal vein (70%) and the hepatic artery (30%) [44]. Four forms of vasculature growth and development of new blood vessels are present in the HCC: co-option, angiogenesis (sprouting), vasculogenesis (BM-EPCs recruitment to increase the tumour vascular supply), and intussusceptive angiogenesis [62]. Sprouting angiogenesis is most commonly described as being morphologically characterised by arterialization of its blood supply and sinusoidal capillarization [5,55,57]. Arterialization (arteriogenesis) is defined as the growth of functional collateral arteries covered with smooth muscle cells from pre-existing arteries [55,62]. Sinusoidal capillarization leads to distortion of their lumens and loss of EC fenestration associated with basement membrane synthesis. Consequently, the process involves the transformation of fenestrated hepatic sinusoids into continuous capillaries, which are characterized by the expression of some markers absent in the normal sinusoidal endothelium [44,55,57]. The process by which new vessels are formed from tumour cells is termed “vasculogenic mimicry” (VM) [63]. The latest works also bring attention to mechanisms of VM in the HCC, a process induced by HIF-1α regulation of Lysyl oxidase-like 2 (LOXL2) expression [32]. 

The participation of the BM-EPC subpopulation in HCC angiogenesis is considered uncertain by one of the authors [5,64] but otherwise, is mostly accepted [28,29,62,65,66]. Different types of BM-derived cells exhibit effects on HCC tumour growth in a coordinated manner by promoting angiogenesis. Hence, BM-derived VEGF-R2-positive CD133+ cells promoted tumour progression at the middle stage of HCC, while BM-derived, CD45+, CD133+ cells were extremely important for metastasis of HCC at the middle and late stages [67]. 

## 4. Endoglin—Characteristics of the Molecule

### 4.1. Endoglin Structure and Best-Known Functions

Endoglin (CD105) is a type I integral membrane-bound glycoprotein that serves as a co-receptor (with betaglycan) for members of the transforming growth factor-β (TGF-β) superfamily of proteins which has a crucial role in fibrogenesis and angiogenesis [24,68,69,70]. TGF-β ligands and receptors make up a complex signalling system which is very well described in the literature [23,41,71,72,73]. TGF-β, as a pleiotropic cytokine, regulates basic cellular processes such as proliferation, migration, adhesion, cytoskeleton organisation, apoptosis, and extracellular matrix reorganisation, which play a key role in carcinogenesis [23,73]. TGF-β, together with hypoxia, include the increase in endoglin gene promoter expression [74]. On the other hand, endoglin (and betaglycan) modulates (inhibits, enhances) TGF-β-dependent responses in several cell types, with some mechanisms of this regulation still being discussed [23,42].

Endoglin is a 180 kDa homodimer and consists of a large extracellular domain (561 aa), regular hydrophobic transmembrane domain, and a short serine/threonine-rich cytoplasmic tail [23,68]. It occurs in two isoforms, long (L) and short (S), differing in the length of cytoplasmic domain (47 aa vs. 14 aa, respectively), cellular localisation, and the level of phosphorylation [11,23,75]. ASF/SF2 is involved in the synthesis and alternative splicing of endoglin, with its overexpression in ECs favouring the synthesis of the S isoform. Short endoglin (S-ENG) is an isoform characteristic for ageing or senescence of ECs [76]. 

In humans, endoglin is expressed in the liver with a much higher abundance of the L-ENG splice variant [13]. The research on the murine model has shown that S-ENG is an anti-angiogenic molecule, in contrast to the pro-angiogenic activity of L-endoglin [77]. Meurer et al. identified a novel form of L-endoglin (S’-ENG) in rats, which may play a role in liver fibrosis [78]. It was demonstrated in a rat myoblast cell line that human L-ENG isoform promotes activation of activin receptor-like kinase 1 (ALK1)/SMAD1/5 signalling and S-ENG—the ALK5/SMAD2/3 signalling. Hence, both isoforms of endoglin exhibit different biological activities. The biological effects of higher intensity were achieved through the use of L-ENG, as compared to S-ENG. The action of the L isoform caused accumulation of the smaller amount of type-I collagen and connective tissue growth factor (CTGF) in myoblast, compared to the control. Meanwhile, S-ENG promoted ECM synthesis through the TGF-β/ALK5 pathway in those cells [79]. Human endoglin protein exhibits an additional tripeptide Arg-Gly-Asp (RGD domain) (374–376 aa) in an exposed region of the extracellular domain, which takes part in recognizing the ECM proteins, as well as cellular adhesion [38,68,80,81]. 

Endoglin also occurs in a soluble form (Sol-ENG), which was detected in the serum of healthy as well as diseased patients (including those diagnosed with HCC) [17,18,19,40,72,82]. Sol-ENG is created by proteolytic cleavage of the extracellular domain of the endoglin by matrix metalloproteinase 14 (MMP14) [83,84]. This form of endoglin is released into the bloodstream not only by ECs, but also by cancer cells that contain endoglin on their surface [23,83]. 

### 4.2. Endoglin and Signalling Pathways 

Endoglin engages in many protein complexes, including those with TGF-β superfamily members: TGF-β1 and TGF-β3 isoforms, type-I and type-II TGF-β serine/threonine kinase receptors (TGF-β RI, TGF-β RII), and other cytoplasmic proteins [23]. Endoglin connects with TGF-β RI, TGF-β RII through the cytoplasmic, as well as extracellular domain [73,85,86]. As mentioned, endoglin serves as an auxiliary receptor for the TGF-β forming, together with betaglycan and CD109 antigen, type III TGF-β receptor (TGF-β RIII) [71,73]. Endoglin, in contrast to betaglycan, only binds ligands when it is associated with TGF-β RII [73]. Endoglin forms stable homodimers that function as a scaffold for TGF-β RII, ALK5, and ALK1 binding. Signalling data showed a role of the quaternary receptor complex in regulating the balance between TGF-β signalling to SMAD1/5/8 and SMAD2/3 [87]. 

Genetic and biochemical studies of quiescent ECs prove that endoglin is necessary for TGF-β/ALK1 (a type I TGFβ receptor, ECs restricted) signalling [73]. In a not completely discovered mechanism, endoglin, through stimulation of TGF-β/ALK1 signal transduction, indirectly inhibits TGF-β/ALK5 (the second subtype of TGF-β RI, broadly expressed) signalling [73,88]. After endoglin joins the TGF-β RI (ALK1 or ALK5) complex, the signals are transported from the cellular membrane to the nucleus via phosphorylation of transcription factors called SMADs. These, in turn, regulate transcription activity of many target genes when translocated to the nucleus. ALK1 activation is responsible for SMAD1/5 phosphorylation, resulting in stimulation of ECs proliferation and migration, whereas ALK5 activation phosphorylates SMAD2/3, inhibiting those processes [71,73,86,88]. It is worth noting that in quiescent non-proliferative endothelium and/or in the absence of endoglin, the TGF-β/ALK5/SMAD2/3 pathway dominates, while in proliferating ECs, TGF-β/ALK1/SMAD1/5/8 is more active [73,88,89]. 

### 4.3. Endoglin and Cellular Effects

Endoglin, similar to other TGF-β receptors, is involved in modulating a response to the binding of TGF-β1 and TGF-β3 isoforms, activin-A, bone morphogenic proteins (BMPs) -2, -7, -9, -10, β-arrestin2, and α5β1 integrin [23,41,73,81,90,91,92,93]. Endoglin-mediated fibronectin/α5β1 integrin and TGF-β pathway crosstalk are important in the promotion of capillary stabilization and developmental angiogenesis in vivo [93]. Rossi et al. found that extracellular domain of human endoglin also promotes specific αIIbβ3 integrin-mediated adhesion of platelets to the endothelium, and may explain some mechanisms of hemostasis, as well as inflammatory or thrombotic changes in the vessels [94]. In mice models, it was shown that endoglin, as a marker of LSECs, plays a role in nonresponsiveness of T cells across MHC barriers. In other words, CD105-positive LSECs have a tolerogenic property [95].

Endoglin may also regulate EC behaviour independently of TGF-β signalling by regulating cytoskeleton organization, protecting from apoptosis, stimulating c-Jun N-terminal kinase 1 (JNK1) phosphorylation, and regulating endothelial nitric oxide synthase (eNOS) expression [73,89]. Endoglin phosphorylation is probably involved in its subcellular localization and, via interaction with ZRP-1 (zyxin-related protein 1), plays a regulatory role in actin cytoskeleton organization [96]. 

### 4.4. Endoglin and the Most Common Diseases

Endoglin gene mutation is responsible for the autosomal dominant type-1 hereditary haemorrhagic telangiectasia (HHT1; Osler-Weber-Rendu syndrome). The most prominent symptoms of HHT1 are capillary dysplasia and recurrent haemorrhage [74], with changes in hepatic vasculature manifesting as large arteriovenous malformations. Liver involvement in the disease associated with endoglin gene mutation concerns over 30% of cases, and might cause liver failure [97]. Molecular changes in domains of the endoglin molecule were thoroughly described [89,98].

Soluble endoglin is also involved in the etiopathogenesis of preeclampsia. In this disease, increased expression of Sol-ENG by the placenta, and a decreased expression of VEGF-A and PlGF cause impaired angiogenesis and vasculogenesis. An intense inflammatory response, EC injury, generalized vascular resistance, and disseminated intravascular coagulation were described as symptoms [89,99]. 

Endoglin is an important marker of angiogenesis in the case of many carcinomas, including HCC [5,41,45,100,101]. Increase in endoglin production is observed mostly in proliferating ECs of small vascular and lymphatic vessels in cancer tissues [40,42,100,102], including HCC [14,20,45,58,103,104]. Increased expression in tumour-associated ECs of small capillary-like vessels most often correlates with increased tumour growth, and shorter DFS and OS, as shown in e.g. colorectal carcinoma [101] or pancreatic tumours [102]. 

Depending on the type of a tumour and endoglin producing cells (vascular ECs vs. tumour cells), this protein can serve dual roles (growth/tumour progression or inhibition/tumour suppression) in carcinogenesis (reviewed in [23,24]). Hence, increased endoglin expression in tumour ECs correlates with increased tumour growth (pro-angiogenic role), and its downregulation is associated with decreased tumour angiogenesis and growth. On the other hand, increased endoglin expression in tumour cells correlates with tumour suppression, whereas downregulation of endoglin leads to tumour progression, allowing migration, invasion and malignancy [23,24]. The deficiency of even a single copy of the endoglin gene in ECs leads to increased metastatic capability due to the weakened EC barrier to tumour cell intra- and extravasation [105]. Moreover, endoglin-deficient ECs displayed features of endothelial-to-mesenchymal transition (EMT) in tumour vasculature, with enhanced signalling through the TGF-β RI/ALK5 pathway [105].

The release of circulating endoglin, Sol-ENG, produced by ECs as well as neoplastic cells, was previously described [17,23,40]. This form of endoglin in tumours serves as a marker of poor prognosis [72]. However, the mechanisms of its influence on tumour growth are still unclear. It is known that it can modulate both the ALK1 and ALK5 signalling pathways [86,88,106]. It was also shown in an in vitro model that Sol-ENG treatment resulted in activation of NF-κB and IL-6, suggesting activation of pro-inflammatory phenotype in HUVECs [107]. 

The most important properties of endoglin, with particular attention to its role in the physiology and pathology of the liver, are presented in Table 1.

### 4.5. Endoglin and Factors Inducing and Inhibiting Its Production

TGF-β1 via SMADs, hypoxia via HIF-1, and vascular injury via Kruppel-Like Factor 6 (KLF6) all upregulate endoglin transcription [84]. Both hypoxia and TGF-βs play a significant role in the development of HCC and angiogenesis. HIF-1, as a main hypoxia transcription factor, activates the genes responsible for angiogenesis, cellular proliferation and invasion, and HCC metastasis [114]. A hypoxia-responsive element (HRE) was also identified in the endoglin gene, causing increased endoglin promoter activity [115]. Hence, both TGF-βs and hypoxia are the main inducers of the endoglin gene promoter [23,74,115,116,117]. A significant increase in the expression of endoglin, ALK1, and phosphorylated SMAD1/5 was confirmed in hypoxic ECs, in both in vitro and in vivo conditions [117]. Another factor that stimulates endoglin production (higher in proliferating than in non-proliferating cells) is dose- and time-dependent irradiation [118]. Vascular damage causes interaction of KLF6 with MMP14, leading to the release of soluble endoglin from ECs [84]. Endoglin production is down-regulated by tumour necrosis factor-α (TNF-α) [116]. 

Other regulators of endoglin expression in various cell types are well summarized in the literature (reviewed in [89]). 

## 5. Endoglin in the Pathogenesis of HCC—Experimental and Clinical Studies

### 5.1. Endoglin and Liver Fibrosis

Numerous studies indicate the participation of canonical and non-canonical TGF-β pathways in the process of fibrosis of various organs (including liver) [11,89,119]. However, the role of endoglin itself in that process (inhibition? promotion?) is still unclear [13,69,120]. Comparative studies of tissue endoglin expression in HCC and precancerous changes indicate the close relationship between the increase in microvessel density (MVD)-CD105 score and liver cirrhosis [12,58]. In contrast to MVD-CD34, the MVD-CD105 score was higher in regenerative nodules (RN) than in dysplastic nodules (DN) and small HCC [12]. Additionally, higher expression of CD105 was detected in the central portion of the RN than in its periphery [58]. Higher endoglin expression levels were also shown in septal myofibroblasts (MFBs) in patients with advanced fibrosis, compared to their localisation in sinusoidal ECs in normal liver [120], as well as active ECs in the liver with focal hyperplastic nodules [113]. 

Experiments on different models (in vitro and in two different in vivo models of liver fibrosis) prove that the endoglin expression increases through the process of HSCs transdifferentiation into MFBs [78,121]. Meurer et al. proved that transient overexpression of endoglin in HSC lines leads to a strong increase in TGF-β-driven SMAD1/5 phosphorylation and α-smooth muscle actin (α-SMA) expression [78]. In other work of that team using the murine HSC model, activation of the SMAD1/5/8 pathway by endoglin, combined with increased phosphorylation of ERK1/2 and overexpression of vimentin, α-SMA, and CTGF, was confirmed. The amount of type-I collagen was, however, reduced [111]. Endoglin deficiency in HSCs significantly worsens fibrosis in response to injury in two different murine models of liver fibrosis and increases α-SMA and fibronectin expression in vitro [13]. The aforementioned research supports the protective role of endoglin produced by HSCs against fibrotic injury, likely through modulation of TGF-β signalling [13]. 

Clinical studies bring up the fact of higher serum Sol-ENG levels in HCC patients with coexisting liver cirrhosis, as well as positive correlations of this protein’s serum levels with serum AFP levels. Some of them even consider Sol-ENG level as an independent marker (Odds Ratio, OR 1.3) for the development of HCC in cirrhotic patients [17]. Higher serum Sol-ENG concentrations, as well as increased tissue expression of that glycoprotein (and TGF-β1), which correlated with histological and serum markers of the fibrotic process, were also detected in patients with chronic HCV infection [122]. In contrast to these results, the study of Prystupa et al. showed the lowest concentration of Sol-ENG in patients with decompensated alcoholic liver cirrhosis and the highest in the control group. Moreover, their study demonstrated that the independent factors affecting the Sol-ENG level were the concentration of bilirubin, INR, and duration of alcohol abuse [123]. 

Summarizing the results of experimental and clinical studies, it seems that endoglin serves mostly a cytoprotective function in the progress of liver fibrosis. At the same time, evaluation of its tissue expression, as well as Sol-ENG serum concentration, might serve to grade the severity of histological changes in livers of patients with an isolated form of fibrosis, as well as fibrosis associated with chronic liver diseases (including HCC). More particular pathomechanisms of liver fibrosis involving endoglin are described in a later part of this work. 

### 5.2. Endoglin as a Tissue Marker of Tumour Angiogenesis in HCC

The main molecules promoting angiogenesis in HCC are well known and described. They involve families of factors such as VEGF, angiopoietins, epidermal growth factors (EGFs), Platelet-Derived Endothelial Growth Factors (PD-ECGFs), or basic Fibroblast Growth Factors (bFGFs) [5,124]. The anti-angiogenic factors, which include e.g. matrix metalloelastase, TSP-1, angiostatin with its precursor- collagen XVIII, TIMP-1 and TIMP-2 are much less researched [5,31]. 

The studies investigating the role of endoglin in carcinogenesis of tumours (including HCC) can be divided into two groups: (1) those focused on roles of endoglin as a marker of angiogenesis and its role in diagnosis/prognostics [16,40,100], and (2) those researching pro- and anti-angiogenic mechanisms of endoglin action, based on in vitro and animal models [16,105]. The works of the former group also evaluate the effectiveness of endoglin as a marker of intratumoral MVD (IMVD), based on the results of the endoglin expression in activated ECs, i.e., the main location of this glycoprotein’s synthesis during the course of tumour angiogenesis [12,14,15,20,21,22,100]. 

In the first group of studies, the newest (and, for now, the only) available meta-analysis of data from 34 works indicates that high MVD-CD105 is a predictor of the poor OS, DFS, and CSS (cancer-specific survival) [125]. However, this analysis does not include HCC. 

Tissue expression of CD105 in vascular ECs (both in sinusoidal ECs in the region of the tumour and blood vessels in the portal areas of non-cancerous liver tissue) was detected by most of the authors [12,14,15,20,21,22]. The results of these studies differ, however, considering the following aspects: (1) detectability (frequency of occurrence) of CD105 immunostaining (positive, negative) in HCC and control liver; (2) location-dependent intensity of CD105 expression (tumour, adjacent non-tumorous tissue, normal liver); (3) type of statistically significant correlations (positive, negative) with clinical advancement of the tumour (TNM staging), prognosis (patient survival time), and other clinical data (e.g. AFP concentration), as well as expression of other pro-angiogenic tissue factors (e.g., VEGF). 

In HCC tissues, three patterns of CD105 expression on ECs, namely, sinusoid-like, branching, and small endothelial sprouts, first described by Ho et al., are most commonly cited [103]. This study describes detection of CD105 expression in tumour tissues in ~70% patients, contrasting with so-called “diffuse” pattern of CD105 expression in the adjacent non-tumorous liver tissues in some HCC cases [103]. However, these authors did not study the expression of CD105 in “normal” (control) liver tissue. In turn, Yang et al. described the expression of CD105 in the vascular ECs of all HCC tissues, but not in the vascular ECs of any normal or paracarcinomatous liver tissue [14]. On the other hand, immunohistochemistry studies by Minhajat et al., showed comparable detectability of expression in blood vessels of portal areas of non-cancerous areas (100% patients, intense IHC reaction in all cases), as compared to CD105 expression in sinusoidal ECs in cancerous areas (80% patients, intense and moderate grade of IHC reaction). It is worth adding that these authors also described weak cytoplasmic expression of CD105 in normal hepatocytes in non-cancerous areas, as well as in cancer cells in the cancerous tissue. These authors also did not include “normal” liver (control) in their studies [45]. A detailed comparison of CD105 expression in heterogeneous regions of the liver, notably, tumour tissue (TT), adjacent non-tumour tissue (AT), tumour free tissues (TF), as well as healthy control liver, can be found in a study by Yu et al. [20]. CD105 expression in endothelial sprouts of tumour tissue of all of the HCC patients, and a diffuse pattern of staining in most cases, was confirmed, predominantly in hepatic sinusoidal ECs in the surroundings of draining veins in AT and TF. Quantitatively, the highest expression (at mRNA and protein levels) concerned the AT region with cirrhosis, followed by TF tissues, as compared to HCC and control. High correlation coefficients were detected between the expression of CD105 and VEGF mRNA, in all of the fragments of studied liver excluding healthy control [20]. Others described the positive expression of CD105 only in the adjacent non-tumorous area with newly-formed vessels [21], or higher MVD-CD105 score in regenerative nodules than in dysplastic nodules or small HCC tissue [12]. The research by Yao et al. described typical patterns of CD105 expression observed in other studies [20,103]. However, the quantitative expression of CD105 in HCC and non-tumour or control liver tissue was not compared. The superiority of CD105 over CD34 as an HCC angiogenic marker, as well as that of “large” paraffin sections over tissue microarray (TMA) for more detailed evaluation of correlation with clinical data (e.g. survival time), were both proven [15]. The results of studies by other authors [20] concerning the positive correlation between CD105 and VEGF expression were confirmed [15]. The research by Ribeiro et al. confirmed the observation of lower CD105 tissue expression in all HCC samples (the mean CD105 percentage = 11.2%), compared to cirrhotic tissues (46.9%). In the HCC regions, significantly higher expression of CD105 in well-differentiated HCC tissue, compared with poorly and moderately differentiated tissue, was observed. However, in contrast to previous observations [15,16,20], these authors observed an inverse relationship between MVD-CD105 and VEGF scores [22]. 

The most recent studies generally confirm the observations concerning higher CD105 expression in NT tissue, as compared with HCC. Considering the TMA (90 patients), elevated expression of endoglin in all of a tumour adjacent tissues was observed, while in HCC only ~56% of the affected differentially expressed CD105 (from abundant, weak to negative reaction). The intensity of CD105 expression was significantly higher in NT tissue compared to HCC. CD105 glycoprotein was mainly located in the ECs. However, similarly to the findings of other authors [45], it was also present in the tumours cells. In the group of patients with negative CD105 expression (~44%), around 75% showed poorly-differentiated HCC. This research was validated on fresh HCC material, proving that the expression of CD105 lowers with progressing severity of the disease [104]. Interestingly, vasculogenic mimicry was also proved as a marker of poor clinical prognosis in HCC. However, it was associated with CD105-negative VM vessel cells. These cells were not derived from ECs but were similar to HCC tumour cells [63]. 

### 5.3. MVD-CD105 as a Prognostic Factor in HCC

Most of the authors agree that the endoglin is an ideal instrument to study HCC microvessel density, i.e., it is better than the use of pan-endothelial EC markers (e.g., CD31, CD34), as it visualises the ECs of newly formed tumour vasculature and correlates with VEGF expression [14,15,16]. Additionally, endoglin staining reduces false-positive staining of blood vessels, compared with other commonly used pan-endothelial markers. Immunohistochemistry can be easily performed on formalin-fixed, paraffin-embedded tissues [15,103]. Experience also shows that in many types of cancer, MVD counted using CD105 is a better estimator of tumour prognosis and survival than MVD counted by pan-endothelial markers [14,15,16].

Similarly to other solid tumours of the gastrointestinal tract [100], HCC also shows positive correlations between increased MVD-CD105 expression and clinical-pathological data (TNM staging, the degree of tumour differentiation, portal vein invasion, and lymph node metastasis) and/or poor prognosis [14,15,16]. Research by Yang et al. point that CD105 may be an independent prognostic marker of HCC survival, as well as an independent prognostic indicator of recurrence and metastasis in HCC patients. At the same time, this study shows that early post-surgery prognosis (2-year survival) of HCC patients with lower CD105-MVD was significantly higher than in HCC patients with a higher CD105-MVD (47.1% vs. 13.5%) [14]. The results by Yao et al., performed on HCC tissue material as well (but not using HCC tissue microarrays), also contain an observation that a higher MVD-CD105 score (as cut-off points) or in patients with HCC significantly correlated with poorer prognosis of the patients (DFS and OS) [15]. On the other hand, the MVD-CD105 score of the tumours investigated by the researchers was lower in larger tumours, more aggressive tumours, and in tumours with more advanced TNM stage [15]. These results are in accordance to the observations of Ho et al., who also described correlations between lower IMVD-CD105 scores and larger sized (>5 cm) and more aggressive tumours, as indicated by venous infiltration, microsatellite nodules, and advanced TNM tumour stage [103]. At the same time, this study has shown that only the expression of endoglin that follows the diffusion pattern in the microvessels of an NT adjacent tissue was a predictive factor of early recurrence [103].

There are also some results that note negative correlations between MVD-CD105 expression in tumour tissue and prognosis in HCC. Comparative studies of CD105 expression between different regions of HCC and control liver have shown that lowered expression of CD105 in ECs of tumour tissue, and more abundant expression in LSECs in NT tissues (including cirrhosis), might have prognostic significance in this type of cancer [12,20,22,104]. Wang et al. observed a high positive expression of CD105 in an adjacent NT area with newly formed vessels. The MVD-CD105 expression and portal vein tumour thrombus (PVTT) were significantly correlated with recurrence of HCC after liver transplantation. Endoglin expression in the NT area also showed a positive correlation with PVTT, TNM staging, and serum AFP level in HCC patients [21]. Studies by Qian et al., also presented lower OS of patients with negative CD105 expression in tumour tissue. The authors conclude that lowered or negative IHC reaction to CD105 in HCC vessels might indicate severing of the disease and HCC progression [104].

The most recent research based on tissues of HCC patients treated with sunitinib (other than sorafenib, multiple tyrosine kinase inhibitor) showed that tissue expression of CD105 in tumour tissue monocytes, as well as a serum concentration of CD105, might serve as an independent predictive factor of the OS and progression-free survival (PFS) of advanced HCC. Interestingly, patients with lower endoglin expression or serum levels responded better to therapy with that drug [126].

### 5.4. Soluble Endoglin and/or Serum Endoglin mRNA Level in HCC as a Complementary Biomarkers

There are not many studies on the role of endoglin as a serum biomarker in HCC. The present results confirm the elevated levels of Sol-ENG in cirrhotic patients (5.8 µg/L) as compared with healthy (3.7 µg/L) or non-liver-disease controls (3.9 µg/L). The highest concentration of serum CD105 (7.4 µg/L) was observed in patients affected with HCC and cirrhosis, compared to liver cirrhosis only (5.8 µg/L) and HCC only (5.0 µg/L). In a group exhibiting the highest serum CD105 levels, serum AFP levels showed a high correlation. These authors consider Sol-ENG as an independent marker (OR 1.3) for the development of HCC in cirrhotic patients [17]. Similar results were obtained by other authors; however, the correlation between Sol-ENG and AFP was not confirmed [18]. On the other hand, serum levels of both biomarkers (Sol-ENG and AFP) were positively correlated with stages of HCC, but not with Child Pugh’s classification. At a 7.5 ng/mL cut-off, the sensitivity of endoglin was 70% and specificity was 65%. Together with AFP, the sensitivity was higher (85%) [18]. 

Increased endoglin and TGF-β mRNA expression were also demonstrated in the serum of HCC patients with coexisting cirrhosis, as well as cirrhotic patients, compared to control (healthy volunteers), but without a major difference between the diseased groups. Endoglin mRNA concentrations correlated positively with TNM clinical stage [19]. In turn, the research by Yang et al., conducted on HCC patients treated with sunitinib, demonstrated a negative correlation between serum CD105 mRNA expression and the advancement of HCC, PFS, and OS. In other words, OS and PSF were significantly elevated in patients with lower blood (and tissue) CD105 expression, compared to those of higher expression of that glycoprotein. Patients exhibiting lower endoglin levels responded better to sunitinib therapy [126]. 

Summarizing the cited research, endoglin overexpression is mostly observed in proliferating ECs of small vascular and lymphatic vessels in cancer tissues, on different stages of solid tumour carcinogenesis. The studies on the potential role of endoglin as a tissue prognostic marker of HCC progression yield varying results.

Some of the publications describe a positive correlation between CD105 expression in tumour tissue and clinical-pathological data and/or prognosis in HCC, similarly to other tumours of the gastrointestinal tract. However, CD105 positive immunostaining in non-tumour tissues (including cirrhosis) and/or control liver was not always detected, or not studied. 

Another group of reports considers the prognostic significance of increased tissue CD105 expression in non-tumour liver tissue (including cirrhosis), and lowered expression in the tissues of a tumour itself. The lower expression of CD105 in HCC, compared to non-tumour tissue and/or control, is usually described in larger, more aggressive tumours with portal veins invasion, poor differentiated HCC, and tumours with the more advanced TNM stage. According to some of the authors, the presence of higher CD105 expression in non-tumour tissue suggests that CD105 is not a good target of anti-angiogenic therapy in HCC, especially in patients with coexisting liver cirrhosis, as it might promote HCC development. 

There are also studies reporting the comparable expression of CD105 in HCC and non-tumour tissue. Lack of significant differences might be caused by an insufficient number of studied samples or by coexisting viral infections, such as HBV, HCV, that modulate the expression of pro-angiogenic factors, including endoglin. The remaining differences in the results of the analysed works are mostly caused by the diversity of technical approaches, variation in tissue pre-treatment protocols, different types of anti-endoglin mAb used in immunohistochemistry, and non-standardized counting methods.

Singular studies are focused on the analysis of serum levels of Sol-ENG and/or CD105 mRNA level in HCC. These works mostly describe the increased concentrations of this form of endoglin in liver cirrhosis and/or HCC, correlating with some clinicopathological data. They suggest the consideration of Sol-ENG as a useful biomarker in HCC diagnostics. However, the sensitivity of Sol-ENG as an independent diagnostic-prognostic marker seems to be relatively weak. 

In prognostics, as a well as anti-angiogenic treatment initiation or effect monitoring, it seems important to connect the cellular level research (complete analysis of all of a tumour and healthy liver regions) with estimation of levels of serum Sol-ENG (or endoglin mRNA) and/or other markers of HCC advancement.

The results of the studies of the cited authors are compiled in Table 2. 

## 6. Endoglin in Pathomechanisms of HCC

### 6.1. Endothelial Progenitor Cells (EPCs) in the Hypoxic Area of HCC

Yu et al., verified the hypothesis that non-tumour (NT) tissue surrounding the HCC might be a hypoxic and highly angiogenic area, into which many more EPCs are recruited and homed [30,31]. It was proven on a murine HCC model that during the development of HCC, a large number of bone marrow EPCs were mobilized into the circulation. Incorporation of these cells into ECs of different types of vessels (sinuses, capillary and great vessels) was observed [66]. Higher concentrations of circulating EPCs in patients with advanced unresectable HCC, as compared to patients with resectable HCC or those with liver cirrhosis, were observed [65]. 

In turn, it was observed in tissue HCC material that the NT region shows high expression of different angiogenic factors (activator and inhibitor molecules) (reviewed in [31]), which are important for the neovascularization, growth, and development of human HCC, as well as for promoting differentiation of EPCs into ECs in this cancer [30,127]. Observations of higher endoglin expression (mRNA, protein) in ECs present in NT tissue (including liver cirrhosis), compared to a tumour, and positive correlations of such expression with clinical advancement or prognosis of HCC, can be found in the literature [12,20,21,22]. 

In conclusion, the above-mentioned research indicates a potential role of EPCs and their differentiation into tumour ECs (expressing CD105 on their surface) in the process of HCC neovascularization. Increased expression of angiogenic factors (including endoglin) in the NT environment suggests that this region of the liver has a key role, especially in initial phases of liver carcinogenesis. 

### 6.2. CD105-Positive Endothelial and Non-Endothelial Cells in HCC

**Figure 1:**
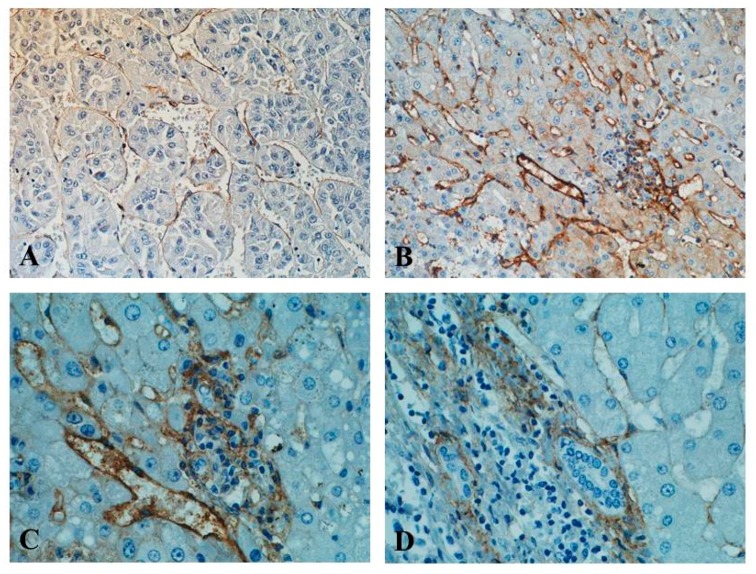
Immunohistochemical expression of endoglin (CD105) as shown by brown staining of the vasculature in HCC samples. (**A**) CD105 immunoexpression in tumour sinusoidal endothelial cells; (**B**) CD105 immunoexpression in tumour microvessels of other patient with HCC (hot spot area); (**C**) higher magnification of the HCC fragment of the same patient, showing ECs and CD105-immunopositive inflammatory cells; (**D**) very weak CD105 localization in peritumoral hepatic tissue and in bile duct cells in portal space. New polymer-based immunohistochemistry with DAB staining. Haematoxylin counterstained. Original magnification ×200 (**A**,**B**); ×400 (**C**,**D**) (our unpublished data).

Participation of endoglin (CD105), as a proliferation-associated endothelial cell adhesion molecule included in the neovascularization process of HCC, is indisputable [4,5,31,62]. Along with HCC progression, at least some ECs undergo phenotypical changes in liver sinusoids, losing characteristic morphological-structural properties of “normal” LSECs, and gaining several markers that are characteristic of capillary ECs [44,128]. One of these markers is, in fact, CD105, which is considered superior to other pan-endothelial markers such as CD34 [15,103]. The cells that produce endoglin in vivo in HCC are, therefore, activated (proliferating) ECs, otherwise called tumour endothelial cells (TECs) [15,21,103,128,129,130] (Figure 1). 

In recently conducted studies on morphology, phenotype, genotype and function of TECs derived from clinical HCC, the expression of numerous vascular markers (including CD105), as well as genes responsible for angiogenesis, were detected. These cells presented endothelial phenotype and morphologic features of ECs, and were capable of tube formation and migration [131]. Differential quantitative endoglin expression in ECs (TECs) in different stages of HCC clinical advancement was shown by numerous studies [15,21,130]. It seems that CD105 expression is the highest in well-differentiated HCC, decreasing all the way to negative values in its poorly differentiated form [104]. This might be linked to the more important role of that glycoprotein in initial phases of HCC angiogenesis, in which endothelial sprouting occurs due to hypoxia and acidosis of the tumour microenvironment, which causes overexpression of HIF-1α and activation of endoglin gene promoter [23,74,115,116,117]. 

The CD105 expression is also demonstrated in normal hepatocytes in the NT area [45], as well as in liver cancerous cells [45,104]. Endoglin is also produced by cultured HCC cells (e.g., HepG2 cells). Their comparison with TECs isolated from fresh HCC tissues showed that there was no similarity of phenotype or function between these two types of cells [16,50,131]. 

A role of other CD105-positive liver cells in HCC pathogenesis cannot be ruled out. These include the previously mentioned HSCs [13], human liver-derived stem cells [49,51], and human adipose-derived stem cells [52]. The former differentiate into myofibroblasts (fibrotic role), with the two latter types able to be induced into functional hepatocyte-like cells. Additionally, it was proven that undifferentiated or differentiated adult-derived human liver stem/progenitor cells produce much larger amounts of CD105, compared to “mature” hepatocytes [49]. Recently, in a murine model, the expression of CD105 was also presented in CD146-positive pericytes in the perivascular regions of the liver, considering that these cells from wild-type liver differentiated into myofibroblasts (fibrogenic role) [53].

### 6.3. Endoglin as a Modulator of Tumour Angiogenesis, Tumour Proliferation, Migration, Invasion and Metastasis

TGF-β, together with its endoglin co-receptor, is engaged in the processes of angiogenesis, which was presented on in vitro models and works with experimental animal models [105,129,130,132]. Warrington et al. proved that endoglin, in its normal levels, acts as a pro-angiogenic molecule and is required for the formation of new blood vessels. It was reported that endoglin antagonises the inhibitory effects of TGF-β1 on human and murine vascular ECs [132]. Further works on the model of cultured TECs isolated from HCC, showed higher endoglin expression and pro-angiogenic properties of these cells, enhanced angiogenic activity, enhanced spontaneous motility, and greater capacity to migrate in response to TGF-β1, compared to “normal” ECs [129,130]. Additionally, Xiong et al., described increased apoptosis resistance and resistance to some chemotherapeutic drugs (e.g., sorafenib) exhibited by TECs [130]. 

Using the model of cancer stem-like cell (CSCs) generated from HCV-infected primary and transformed human hepatocytes, it was shown that endoglin is upregulated (250-fold) in sphere-forming cells compared to primary hepatocytes [133]. The same group of researchers observed endoglin expression and activation of phospho-SMAD1/5 and DNA binding protein 1 (ID1) downstream signalling molecules in the HCV core-expressing cell surface of human hepatocyte origin [133]. However, the study of Mardomi et al. proved that the application of CXCR4 and endoglin antagonists in combination with TGF-β, to BM mesenchymal stem cells, decreased the migration rate of BM-MSCs toward HepG2 cells. The study was conducted with the use of an in vitro migration assay [134]. 

There is also some proof of a link between hypothyroidism and elevated risk for HCC. In the endoglin gene promoter, the presence of thyroid hormone response elements (TREs) was also reported [50]. The study of HepG2-TR-expressing cells in vivo, and in vitro proved that the thyroid hormone T3/thyroid receptor (TR) signalling suppresses cell proliferation by up-regulating endoglin and affecting p21 stability. Hence, it seems that endoglin has a suppressor role to inhibit cell proliferation in the HCC cell lines [50]. On the other hand, more recent studies by the same group on human HCC SMMC-7721 cell line reported that CD105 promotes the invasion and metastases of liver cancer cells by increasing VEGF expression [16]. 

Modulatory influence of endoglin on the inflammatory process was also described. In the murine model, the regulatory role of endoglin in leukocyte transendothelial migration, facilitated by binding to the α5β1 integrin (via its RGD motif) after stimulation by the CXCL12 inflammatory chemokine, was also proven. Leukocyte adhesion was inhibited by Sol-ENG and synthetic RGD peptides [81]. This research, while not concerning HCC, also indicates the multiple roles of endoglin molecule domains. Following from this, membrane-bound endoglin shows pro-angiogenic activity [24,93], whereas RGD-containing Sol-ENG displays an anti-angiogenic function [81]. 

**Figure 2:**
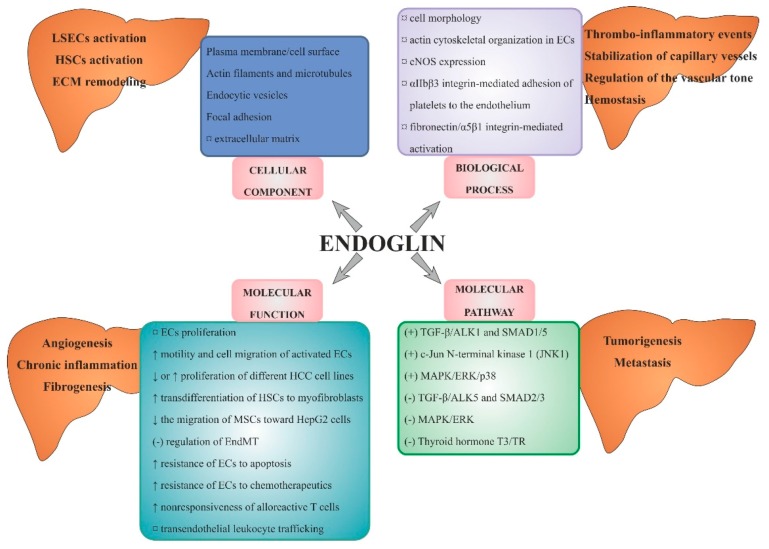
Summary of the roles and signalling pathways of endoglin in HCC angiogenesis and tumorigenesis. Legend: (+)/(−)—stimulation/inhibition; ↑/↓—increasing/decreasing; ¤—regulation; ECs—endothelial cells; ECM—extracellular matrix; EndMT—endothelial-to-mesenchymal transition; eNOS—nitric oxide synthase; HCC—hepatocellular carcinoma; HSCs—hepatic stellate cells; LSECs—liver sinusoidal ECs; MSCs—mesenchymal stem cells; see text for details.

In summary (Figure 2), the research on endoglin role in mechanisms of angiogenesis in HCC proves that, similar to many other tumours, this marker is one of the most optimal markers of neovascularization. Endoglin is involved in the process of arterialization and sinusoidal capillarization in HCC, and might serve an equally important pro-angiogenic in HCC as VEGF. Apart from the ECs, a range of other cells involved in angiogenesis in this type of cancer, which is also characterised by positive endoglin expression, was described. However, identifying the roles of those cells requires further research. 

Considering the fact that HCC is one of the most vascularised solid tumours, evaluation of angiogenesis with the use of anti-endoglin mAb should be included in the routine diagnostics and prognostic procedures in this type of cancer. Evaluation of the MVD-CD105 in tissue material should become an inherent step in the staging of HCC. 

Finally, liver fibrosis (exhibiting CD105 overexpression in HSCs) and HCC (with CD105 overexpression in TECs) can be added to the list of potential therapeutic target diseases for CD105-specific therapy.

## 7. Endoglin and a New form of Anti-Angiogenic Therapy in HCC

Effective cancer therapies should be directed towards not only lowering/inhibition of pro-angiogenic factors, but also target factors of vascular functions [135]. Anti-endoglin mAb established by Tan et al. and passively transfused through tail veins into two murine hepatoma models, effectively suppressed tumour growth, inhibited angiogenesis, increased cell apoptosis within the tumour tissue, and prolonged the survival rate of these mice [136]. Different types of anti-endoglin mAbs were tried, including radiolabelled antibodies or immunotoxin-conjugated antibodies. Recently, some attempts have been made to apply anti-angiogenic gene therapy for cancer, i.e., endoglin silencing by RNA interference, as reviewed in [137]. In the animal model, it was also shown that the endoglin-targeting liposomes can represent new strategic tools for future application of endoglin-directed neoplastic and anti-angiogenic therapies [138]. 

In the case of HCC, the stage of tumour advancement, type of vascularization, and molecular type of angiogenesis are all important parameters. A further challenge will be to find the right combination of anti-angiogenic/anti-vascular drugs and standard forms of radio- or chemotherapy [26]. Previous effects of anti-angiogenic therapies, including embolization of HCC, have not reached expectations. However, new forms of this type of treatment are currently introduced, including trans-arterial radioembolization (TARE), as reviewed in [139]. 

One of the new ideas of HCC therapy is the use of a chimeric IgG1 anti-CD105 monoclonal antibody (TRC105) that binds human endoglin with high avidity. It inhibits angiogenesis and tumour growth by ECs growth inhibition, antibody-dependent cellular cytotoxicity (ADCC) and apoptosis, and complements VEGF inhibitors [10,140]. Data about use of such treatment in animals and humans are inconsiderable. 

### 7.1. Animal Studies 

One of the increasingly used methods of solid tumour therapy the is the administration of cytotoxic radioisotopes conjugated to antibodies. Studies on the treatment with endoglin-targeted radioimmunotherapy have been carried out on a murine model of HCC. HCC tumour SMMC7721-GFP xenograft-bearing mice were treated with ^131^I-anti-endoglin mAb. The effects of this therapy included significant tumour-growth suppression and the remarkably decreased tumour weight in animals [141]. New treatment options are still being sought. One of them is a combination of TRC105 with sorafenib. Such therapy showed the largest reduction of tumour volume in BALB/c mice after inoculation of a murine HCC cell line when compared with sorafenib only or control [142]. Also, results of combined radioimmunotherapy with ^131^I-anti-endoglin mAb and 5-fluorouracil on the same HCC model appear to be promising. The tumour volume and weight decreased, and inhibition rate of the tumour growth was up to 77.1 ± 4.06% [143]. 

### 7.2. Human Studies

The first human study TRC105 treatment was published in 2012. Patients with advanced cancers showed the safety profile and clinical effectiveness of TRC105 therapy [144,145]. Also, combined treatment with bevacizumab proved to be effective; some patients experienced a reduction in solid tumour volume, the others remained without progression for a longer period than during their previous VEGF inhibitor therapy [146]. Sorafenib, the multikinase inhibitor, improved survival in advanced HCC, and is so far the only approved first-line drug for systemic therapy in such cases. TRC105 was used in patients with advanced HCC and compensated liver cirrhosis (Child-Pugh A/B) after ineffective sorafenib treatment, but results were not satisfactory. Only one out of the 11 patients enrolled in the study showed a response to the treatment [147]. In the other study done by the same authors, in HCC patients, mostly with liver cirrhosis and HCV infection, the overall response rate to therapy with TRC105 and sorafenib at all 4 dose levels was 21%. Most of the responders occurred at the highest TRC105 dose level. Duration of the response to therapy ranged from 4.4 to 27.6 months. The study resulted in a median PFS time 3.8 months and median OS 15.5 months in all enrolled patients [142]. 

New combinations of therapies, using TRC105 and axitinib or TRC105 with bevacizumab, are undergoing clinical trials in other cancers (renal carcinoma) [148,149]. Furthermore, TRC105 was shown as an effective radiation sensitizer in the prostate cancer xenograft animal model [150]. Based on the above observations, clinical trials on the use of TRC in various drug combinations in patients with HCC may be expected in the coming years. 

In summary, phase-I and -II clinical trials showed the insufficient efficacy of TRC105 monotherapy in HCC. While still unsatisfactory, better results were obtained in combination therapy of TRC105 with sorafenib. On the basis of preclinical tests and data from the other types of tumours, it may be expected that TRC105 therapy in combination with other promising agents, evaluated in phase-III trials as first-line and second-line treatment for HCC, will yield better results. Knowledge of liver sinusoidal ECs markers (including CD105) helped to identify novel targets of anti-angiogenic therapies and/or to understand adverse effects of some drugs used for such therapy [2,44]. Due to the high heterogeneity of pathological changes in HCC, as well as the heterogeneity of tumour evolution, an effective systemic therapy of this cancer is not yet available; this remains a goal of the precision medicine development. 

### 7.3. Nanoparticles as a Potential Way of HCC Therapy

The therapy of various cancers with the use of nanoparticles (NPs) has become a current focus of interest. The combination of these unique NPs, possessing different physical and chemical properties, with other substances or drugs, increases the efficacy and safety of anticancer therapies compared to conventional methods. NPs can be designed to achieve the physicochemical and biological properties that are necessary to reach tumour cells while saving the healthy surrounding tissue. The data describing NPs anticancer activity in HCC in vitro and in vivo are not abundant in quantity, in contrast to those of other cancers [151,152]. 

Eco-friendly gold NPs (metal NPs), synthesized by Enterococcus spp. bacteria showed cytotoxic activity against HepG2 cell line in vitro. The anticancer activity depends on the concentration of NPs, the higher one gives the higher activity. In this study, the high efficacy was compared to standard cyclophosphamide [153]. Silver NPs, TOL-AgNPs, derived from common dandelion (Taraxacum officinale), showed high cytotoxic activity against Hep-G2 cells [154]. The other metallic oxide NPs, injectable NBTXR3 hafnium oxide NPs, are under evaluation in phase I/II clinical trial, together with stereotactic body radiation therapy (SBRT), in HCC or liver metastasis cases. First results of this study show that NBTXR3 stays within the tumour without negative effects on liver functions or the reliability of the image-guided radiation therapy [155]. 

Non-metallic silica NPs were used to improve the effective therapy of liver cancer. The functionalized silica nanoparticles (SLNs) were conjugated with doxorubicin via disulphide bonds, using a CPLGLAGG peptide substrate which may be split by the MMP-2 protease. In vitro and in vivo studies in HepG2 cells and HepG2 tumour-bearing mice confirmed that overexpressed MMP2 protease in tumour tissue, together with intracellular glutathione, causes rapid release of doxorubicin, leading to inhibition of tumour growth [156]. The low-density lipoprotein (LDL) modified silica NPs were used to deliver drugs such as docetaxel (DTX) and thalidomide (TDD) to HepG2 cell line. Targeting LDL receptors, overexpressed on HepG2 cell line, by nano-sized LDL/SLN/DTX/TDD increased efficacy anti-cancer effects [157]. Other lipid NPs were also used to increase the chemotherapeutic effect in xenograft-bearing mice. SP94-conjugated PEGylated liposomal doxorubicin (SP94-LD) presented a significant increase in drug accumulation in tumours, while its plasma residence time was the same as that of its non-targeted equivalent. This confirms that peptide SP94 improved therapeutic results of liposomal doxorubicin in a xenograft mouse model through enhanced tumour apoptosis and decreased angiogenesis [158,159]. 

In HCC cases, the association of NPs with anti-CD105 antibodies is only applicable to tumour diagnostics. The first data about anti-CD105 antibodies conjugated with gold nanoparticles was presented in 2014 in the trial of biodistribution of this complex in mice tumours. There was no observed effect on CD105-dependent tumour uptake and the efficacy of tumour targeting [160]. The combination of CD105 and NPs was used to investigate the proliferation inhibition and apoptosis promotion in MHCC-H and HepG2 hepatoma cells as a method of cell destruction. CD105-labelled docetaxel-loaded lipid microbubble (CD105-DLLM) exerted influence on MHCC-H and HepG2 hepatoma cells during ultrasound triggered microbubble destruction (UTMD). Western-blot analysis showed downregulated expression of proliferating cell nuclear antigen and apoptosis proteins, such as Caspase-3, with up-regulated expression of ERK1/2 and p38 protein in MHCC-H cells, which proves the activation of MAPK signal transduction pathway [161]. 

In summary, reports describing the use of NPs in HCC therapy are not abundant. However, they indicate that the combination of anticancer drugs with NPs may be a sensible method for drug delivery, as well as prevention of drug resistance. However, these require confirmation in clinical trials in human HCC cases. Such experimental therapies are now only in the initial design phase and require a long time to reach the potential widespread use. No data was found in the literature on HCC therapy that combines anti-CD105 agents with NPs and other drugs.

### 7.4. Summary and Perspectives

Regulation of the angiogenic process in HCC, as well as other solid tumours, mainly involves cells that produce pro- and anti- angiogenic factors. Endoglin, as an important TGF-β co-receptor, serves its main role as a pro-angiogenic factor, responsible for the proliferation and survival of ECs (anti-apoptotic action). 

Endoglin expression also positively correlates with VEGF. However, endoglin is expressed on a much higher level (up to 3 × 10^6^ copies per cell) than the VEGFRs (<0.2 × 10^6^ copies per cell) [162]. Endoglin, as an antigen, also proved to be effective as a unique immunohistochemical marker of activated ECs. Visualisation of positive CD105 expression can be easily performed on formalin-fixed, paraffin-embedded tissues, which has a major practical significance. MVD evaluation with the use of anti-endoglin antibodies should become a part of the routine staging of HCC.

The so far obtained results on the expression of endoglin in HCC are relatively sparse, and are often contradictory. Statistically-significant correlations were described between both increased and decreased levels of MVD-CD105 in the tumour tissue and degree of HCC differentiation, more advanced clinical stage of HCC, and/or poor prognosis. Comparing the quantitative expression of endoglins in tumour tissue and non-tumour tissue, the higher expression in microvessels of non-tumour tissue (including liver cirrhosis) (NT) was often noted. Some researchers consider the latter location as a high angiogenesis area, a niche of EC progenitors, or the location of HSC activation towards myofibroblasts; in short, a unique microenvironment for tumour metastasis and growth. Others attribute the high expression in NT tissue to the protective functions of the endoglin, pointing out that anti-CD105 therapy could further damage the liver in early stages of the disease. 

In vivo and in vitro studies of endoglin action mechanisms in HCC point out that in the liver, the effects of this protein are cell-specific, depending on the aetiology of HCC (e.g., higher expression in chronic hepatitis C), HCC differentiation, and stage of advancement of the pathological changes (e.g., higher expression in HCC with cirrhosis). It could be confirmed that endoglin has pro-angiogenic effects in tumour ECs, while at the same time promoting or inhibiting the processes of liver fibrosis and carcinogenesis when produced by other cells. 

Endoglin was chosen as a target for anti-angiogenic therapy, based on the observations of overexpression of this glycoprotein mainly in the newly sprouted tumour blood vessels, and not in the healthy vessels in the rest of the organ. The superiority of anti-angiogenic strategies over standard chemotherapeutics is often stated. In the case of TRC105 use, a few side-effects of such therapy were reported. However, this topic was never brought up in the studies of HCC [42]. Despite that fact, the results of the individual clinical trials that employed anti-endoglin monotherapy in HCC are far from satisfactory. The causes of that fact, for now, can only be speculated. It is known that all forms of anti-angiogenic therapy can potentially interfere with physiological angiogenic processes (e.g., regeneration after injury). This is especially important in the case of the liver, an organ supported by two major vessels [42]. Additionally, HCC is a very heterogeneous tumour, with multifactorial aetiology, which makes it a hard target for targeted anti-angiogenic therapy. New strategies of such methods of treatment appeared recently (e.g., nanoparticle-based treatment). However, they are still in the initial phases of studies in HCC, or have not yet been applied to this type of cancer.

In future research joining the fields of basic sciences and biotechnology, it could be worth analysing the EC progenitors in the liver or determining more precisely of the roles of other cells that synthesise endoglin in hepatocarcinogenesis (including tumour cells). 

Additionally, to determine the quantitative expression of endoglin in different regions of the affected liver more precisely, verification of quantitative methods of MVD-CD105 evaluation, as well as the applied study protocols, is still needed. From a clinical point of view, the qualification criteria for patients set to undergo anti-angiogenic HCC therapy should be more particular and monitored using better methods, especially in those affected by co-existing chronic conditions (e.g., cardiovascular diseases) in which endoglin and TGF-β signalling pathways play a major role. 

## 8. The Main Headlines of the Review and Conclusions

- Endoglin (CD105) is involved in the process of HCC neoangiogenesis (arterialization and sinusoidal capillarization) under intratumoral hypoxia and acidosis, the main important characteristics of the tumour microenvironment.
- Endoglin is expressed preferentially on activated liver sinusoidal ECs (LSECs) which are characterized by enhanced angiogenic activity, spontaneous motility, greater capacity for migration, and increased resistance to apoptosis in response to TGF-β1.
- In HCC, three patterns of the CD105 expression in ECs are most commonly described (sinusoid-like, branching, and small endothelial sprouts).
- Both CD105-positive LSAECs and CD105-positive non-ECs (normal and cancerous hepatocytes, hepatic stellate cells, human liver-derived stem cells/progenitor cells, human adipose-derived stem cells) play a role in the pathogenesis of HCC.
- The large spectrum of endoglin effects in the liver is cell-type and HCC stage-specific.
- High expression of endoglin in non-tumour tissue suggests that the microenvironment might play an especially important role in the progression of HCC.
- For a reliable assessment of the diagnostic and prognostic role of endoglin in HCC, simultaneous examination of different tumour areas, with a comparison to healthy control, unification of research protocols, and verification of MVD-CD105 counting systems, are required.
- Endoglin (mRNA, protein) tissue expression level examination should be combined with measurement of serum levels of Sol-ENG and other HCC biomarkers and/or determination of the histological stage of the tumour, to successfully predict HCC and effectively apply anti-CD105 therapy.
- Due to the high heterogeneity of pathological changes in HCC and tumour evolution, the effective anti-CD105 therapy in this cancer is not yet available, remaining a goal of the precision medicine development.

## 9. A Few Key Points

- The main mechanism that stimulates endoglin production in the HCC is the hypoxia of the tumour environment. However, influence of other factors was also proven (e.g., HCV infection, alcohol abuse).
- In HCC-associated angiogenesis in vivo, endoglin expression mainly concerns tumour ECs (TECs), activated ECs in adjacent non-tumour area, and different non-endothelial cells (e.g., hepatic stellate cells).
- In HCC, there is a tight relation between overproduction of endoglin by proliferation tumour ECs (TECs) and creation of sinusoid-like, branching, and small endothelial sprouts. The same relation is not observed between endoglin expression and vasculogenic mimicry.
- Higher endoglin expression in non-tumour area, as compared with tumour tissue, suggests that this location may play a very important role in the progression of HCC.
- The large spectrum of endoglin effects in the liver is cell-type specific, depends on aetiology (higher expression in HCV infection), and is HCC stage-specific (higher expression in HCC with cirrhosis).
- Due to the heterogeneity of HCC and multifactorial aetiology of the cancer, the satisfactory effects of anti-CD105 monotherapy (TRC105) are not yet available, remaining a goal of the precision medicine development.

## Figures and Tables

**Table 1:** The molecular structure of the endoglin (CD105), cellular sources in the liver, and the principal role in the liver physiology and pathology.

Criteria	Characteristics	No. of Ref.
Gene Location and size (kb)	Chromosome 9q34.11; ~40 kb; 14–16 exons, human gene encodes 15 exons	[40,41,42,108,109]
Class of Genes	Protein coding; Zona pellucida family; CD molecules	[98,109]
No. of Transcripts	3	[109]
Protein m.w. (kd), no. of Amino Acids	Homodimer, ~180 kD; 2 subunits 95 kDa disulfide-linked; 633-658 aa	[38,42,68,75,81,98]
Cellular Sources in Liver	Quiescent ECs in sinusoids, arterioles and venules	[23,39,40,41,42,43,100]
	Activated LSECs or TECs	
	Normal and cancerous hepatocytes	[44,45,49]
	HCV core-expressing human hepatocytes	[70]
	Myofibroblasts	[78]
	Hepatic stellate cells	[11,13,78,110,111]
	Hepatic perivascular mesenchymal stem cells	[48,53]
	Pericytes	[53]
	Adipose-derived stem cells	[52]
	Adult-derived human liver stem/progenitor cells	[49,51]
Main Role in Liver Physiology	Angiogenesis and vascular development	[24,39,40,85,88]
	Vascular homeostasis	
	Maintenance of vessel wall integrity	
Main Role in Liver Pathology	Stimulation of tumour neovasculature	[88,89]
	Vascular remodelling/modulation of vascular tone	[24,88,89]
	Fibrosis (stimulation/inhibition)	[11,69,89,112,113]
	Diagnostic biomarker in HCC	[17,18,22]
	Risk factor of HCC in patients with liver cirrhosis	[17,19]
	Predictor of HCC early recurrence/distant metastasis	[14,103]
	Risk factor for post-transplant recurrence	[21]
	Predictor of decreased survival in HCC	[14,15,104]

Legend: CD—cluster of differentiation; ECs—endothelial cells; HCV—hepatitis C virus; HSCs—hepatic stellate cells; LSECs—liver sinusoidal endothelial cells; MFBs—myofibroblasts; TECs—tumour ECs; no. of ref.—number of references.

**Table 2:** Endoglin (CD105) tissue expression and serum levels in patients with HCC, non-tumour liver changes (including cirrhosis) (NT) and healthy liver (C).

Tissue Expression			
C	NT	HCC	No. of Ref.
nt	(+); Variable pattern with diffuse expression in some cases #	(+) (68% of HCC); 3 patterns of expression; ↓score ♣,♦	[103]
(−)	(−)	(+) (100% of HCC) ↑score ♦,#	[14]
nt	(+) (100% cases); (+) in “normal” hepatocytes	(+) (80% of HCC); (+) in some cancer cells	[45]
(+)	↑score; typical pattern	(+); typical pattern	[20]
nt	nt	(+) (90% of HCC); ↓score ♣,♦ ↑score#	[15]
nt	↑↑↑score ♦,#	(−)	[21]
nt	↑score in RN>DN>HCC	(+)	[12]
(+)	nt	↑score ♣,♦	[16]
nt	↑score	(+); ↓score ♣	[22]
nt	↑score (100% cases)	(+) (57% of HCC) ↓score ♣,#	[104]
**Serum Levels**			
(+)	↑↑	Sol-ENG ↑↑↑ (HCC + cirrhosis) *	[17]
(+)	↑	Sol-ENG ↑↑,♦	[18]
(+)	↑↑	mRNA-ENG ↑↑,♦ (HCC + cirrhosis)	[19]

Legend: Sol-ENG—soluble endoglin; mRNA-ENG—endoglin transcripts; (+)/(−)—positive/negative expression in tissue/serum; ↑/↓—significant increased/decreased of endoglin expression as related to control or between HCC and NT; ♣—association between endoglin expression with degree of HCC differentiation; ♦—association between endoglin expression and more advanced clinical stage of HCC (TNM, tumour size, venous infiltration, microsatellite nodules, metastases); #—significant correlation with poor prognosis (DFS, OS, etc.) and/or cancer recurrence; *—significant correlation with AFP level; RN—regenerating nodules; DN—dysplastic nodules; HCC—hepatocellular carcinoma; nt—no tested; no of ref.—number of references in order to citation (for details, see text).

## Abbreviations

aa	Amino Acids
ADCC	Antibody-Dependent Cellular Cytotoxicity
AFP	Alpha-Fetoprotein
ALK1/2/5	Activin Receptor-Like Kinase 1/2/5
ASF/SF2	Alternative Splicing Factor/Splicing Factor-2
BMPs	Bone Morphogenetic Proteins
CD	Cluster of Differentiation 31, 34, 105, etc.
CSS	Cancer-Specific Survival
CTGF	Connective Tissue Growth Factor
CXCL12	C-X-C Motif Chemokine 12 or Stromal Cell-Derived Factor 1 (SDF1)
DFS	Disease-Free Survival
ECs	Endothelial Cells
ECM	Extracellular Matrix
EGF/R	Epidermal Growth Factor/Receptor
EndMT	Endothelial-to-Mesenchymal Transition
ENG	Endoglin (CD105)
eNOS	Endothelial Nitric Oxide Synthase
EPCs/BM-EPCs	Endothelial Progenitor Cells; Bone-Marrow EPCs
ERK1/2	Extracellular Signal-Regulated Kinase 1/2
FGF/bFGF	Fibroblast Growth Factor/Basic FGF
HBV/HCV	Hepatitis B/C Virus
HCC	Hepatocellular Carcinoma
HHT1	Hemorrhagic Teleangiectasia Type 1
HIF	Hypoxia-Inducible Transcription Factor
HRE	Hypoxia-Responsive Element
HSCs	Hepatic Stellate Cells
HUVECs	Human Umbilical Vein ECs
ID1	DNA Binding Protein 1
IHC	Immunohistochemistry
IL	Interleukin
INR	International Normalized Ratio of Prothrombin Time of Blood Coagulation
KLF6	Kruppel-Like Factor 6
L-ENG	Long-ENG
LOXL2	Lysyl Oxidase Like-2
LSECs	Liver Sinusoidal Endothelial Cells
MAPK	Mitogen-Activated Protein Kinase (Originally Called ERK)
MMP2, -14	Metalloproteinase 2, -14
MVD/IMVD	Microvessels Density/Intratumoral MVD
NF-κB	Nuclear Factor Kappa-Light-Chain-Enhancer of Activated B Cells
OR	Odds Ratio
OS	Overall Survival
PD-ECGF	Platelet-Derived Endothelial Cell Growth Factor
PDGF/R	Platelet-Derived Growth Factor/Receptor
PFS	Progression-Free Survival
PIGF	Placental Growth Factor
PVTT	Portal Vein Tumour Thrombus
RGD domain	Arg-Gly-Asp Domain
S-ENG	Short ENG
αSMA	Smooth Muscle Actin α
SMAD	The Main Signal Transducers for Receptors of TGF-β Family
Sol-ENG	Soluble Endoglin
TAF	Tumour-Angiogenesis Factor
TARE	Transarterial Radioembolization
TECs	Tumour Endothelial Cells
TGF-α, β	Transforming Growth Factor α, β
TIMP1/2	Tissue Inhibitors of Metalloproteinase 1 and 2
TNF-α	Tumour Necrosis Factor α
TNM	Classification of Malignant Tumours (T—tumour; N—lymph nodes; M—metastasis)
TRC	Anti-CD105 Monoclonal Antibody
TRE	Thyroid Hormone Response Element
TSP-1	Thrombospondin-1
VEGF/R	Vascular Endothelial Growth Factor/Receptor
ZRP-1	Zyxin-Related Protein 1
